# A Systematic Review of Black People Coping With Racism: Approaches, Analysis, and Empowerment

**DOI:** 10.1177/17456916221100509

**Published:** 2022-08-25

**Authors:** Grace Jacob, Sonya C. Faber, Naomi Faber, Amy Bartlett, Allison J. Ouimet, Monnica T. Williams

**Affiliations:** 1 School of Psychology, University of Ottawa; 2 Bioville GmbH, Leipzig, Germany; 3 Department of Psychology, Bryn Mawr College; 4 Department of Classics and Religious Studies, University of Ottawa

**Keywords:** Black people, racism, racial discrimination, emotion regulation, coping

## Abstract

This article reviews the current research literature concerning Black people in Western societies to better understand how they regulate their emotions when coping with racism, which coping strategies they use, and which strategies are functional for well-being. A systematic review of the literature was conducted, and 26 studies were identified on the basis of a comprehensive search of multiple databases and reference sections of relevant articles. Studies were quantitative and qualitative, and all articles located were from the United States or Canada. Findings demonstrate that Black people tend to cope with racism through social support (friends, family, support groups), religion (prayer, church, spirituality), avoidance (attempting to avoid stressors), and problem-focused coping (confronting the situation directly). Findings suggest gender differences in coping strategies. We also explore the relationship between coping with physical versus emotional pain and contrast functional versus dysfunctional coping approaches, underscoring the importance of encouraging personal empowerment to promote psychological well-being. Findings may help inform mental-health interventions. Limitations include the high number of American-based samples and exclusion of other Black ethnic and national groups, which is an important area for further exploration.

Black people in Western societies experience mental and physical stress because of racialization. According to social norms in the United States and Canada, a person racialized as Black (which includes the subcategories African American, Black American, and Black Canadian) may originate from any country. Persons racialized as Black typically share darker skin shades; however, they may have any shade of skin. “Black” here refers to racial grouping, which, in the United States, is defined by government census and is not the same as ethnic group and not synonymous with biological relatedness. Black is a social category, and a person racialized as Black in one country may not be considered Black at all in a different country. For the purposes of this article, Black refers to people in U.S. and Canadian society who are assumed to have African ancestry based on their appearance.

Racial discrimination occurs when a person is mistreated because of their perceived race or ethnic group (Haeny et al., 2021). A person who is regularly exposed to racial discrimination must integrate coping mechanisms into their everyday life to combat the many and ongoing adverse effects associated with race-based stress and trauma. There are many forms that racism can take, and it can occur on individual, community, and institutional levels (Harrell, 2000; J. M. Jones, 1972, 1999). Studies have identified a high prevalence of racist incidents faced by Black Americans, including Lee and colleagues (2019), who found that 69.5% have experienced racial discrimination from time to time or regularly. In the United States, studies have found that Black adolescents cope with incidents of racial discrimination on average five times per day (English et al., 2020). Likewise, Cénat and colleagues (2021) recently demonstrated that at least four of every 10 Black Canadians experience racial discrimination on a weekly basis (Cénat et al., 2021).

Specific mechanisms are required by Black persons to cope with the onslaught of everyday racism, without which they would leave themselves open to significant stress and risk of traumatization, all of which can lead to self-destructive and psychologically taxing responses that may have serious ramifications on a mental and physical well-being (Paradies et al., 2015; Williams et al., 2021). Persistent experiences of racism can lead to an increase in depressive symptoms (English et al., 2020; Wheaton et al., 2018), posttraumatic stress disorder (Helms et al., 2012; Sibrava et al., 2019), and anxiety (Soto et al., 2011). This persistent exposure can also lead to an increased risk of long-term physical illness (Thames et al., 2019), obesity (Sewell, 2017; Stepanikova et al., 2017), diabetes (Bacon et al., 2017; Sewell, 2017), high blood pressure (Brondolo et al., 2011; Forde et al., 2020; Sewell, 2017), and poor birth outcomes (Alhusen et al., 2016; Mustillo et al., 2004).

A Black person may resort to various methods to continue functioning in a systemically anti-Black, racist society. A major mechanism utilized to cope with this aforementioned stress is a set of psychologically defined processes called “emotion regulation” and “coping.” Although these concepts are distinct, they do share some similar characteristics. This article describes the role of emotion regulation and coping as protective responses of Black Americans to race-related stress. Through a systematic review of the literature, we explore the gender differences in how Black people in racialized societies react to racist incidents and then provide recommendations and suggested guidelines for addressing these incidents.

## Coping

Coping has been described as an individual’s changing cognitive and behavioral efforts to manage external and internal demands that are experienced as stressful or that exceed the person’s resources (Lazarus & Folkman, 1984). The coping process is enlisted to respond to stress, and these strategies can change over time and vary depending on the context. Furthermore, there are a multitude of different ways to cope. Skinner and colleagues (2003) identified and assembled 400 different ways of coping.

According to Bridges and Grolnick (1995), all emotion regulation is a form of coping; however, coping involves attempts to regulate one’s emotions specifically in response to a stressful event (for a review, see Skinner & Zimmer-Gembeck, 2007). Many times in a day, people are subjected to different types of stimuli that require them to regulate their emotions (Gross, 1998; Gross & Thompson, 2007; Mauss et al., 2007; Thompson, 1994); however, emotion regulation has generally been defined as the efforts people make to influence which emotions they have the moment they have them, as well as the manner in which the emotions are experienced and expressed (Gross et al., 2006). Typical strategies that are commonly used in emotion regulation include problem-solving, mindfulness, acceptance, distraction, reappraisal, rumination, worry, behavioral avoidance, expressive suppression, and experiential avoidance (Brockman et al., 2017; Naragon-Gainey et al., 2017).

An extensive body of research suggests that some strategies are more adaptive than others. In a large meta-analysis, Webb and colleagues (2012) found that expressive suppression often leads to more negative and less positive emotional experiences, as evidenced by both subjective report and physiological measures. Further, compared with women who smiled in response to distress, women who suppressed their anxiety were rated more negatively on interpersonal characteristics such as warmth and likeability (Bahl & Ouimet, 2022). Finally, people with mood and anxiety disorders report more avoidance, rumination, and expressive suppression along with less problem-solving and cognitive reappraisal (for a meta-analysis, see Aldao et al., 2010). Taken together, these findings suggest that although emotion regulation can have positive short-term benefits by enabling people to cope in the moment, the long-term negative consequences of some strategies may be severe. Understanding how Black people regulate their emotions to cope with race-related stress is thus vital to mitigate the potential downstream harmful effects not only of racism but also potentially the emotion-regulation strategies themselves.

Coping and emotion regulation have common elements that include controlled efforts, intentional efforts, and regulation processes, which have a specific temporal duration (Compas et al., 2014). One important difference between coping and emotion regulation, however, is that coping is uniquely for managing stress. In this systematic review and analysis, we examine how Black people use emotion regulation and other strategies to cope with race-related stress.

## Racial stress requires a unique approach to coping

In the Western world, life experiences differ vastly on the basis of appearance. One of the first well-known psychological experiments carried out through the lens of Black-White racism was by John Howard Griffin, a White journalist who dyed his skin Black and shaved his head to experience typical Black American life in the American South in 1959. He described his everyday experiences in his bestselling book *Black Like Me* (J. H. Griffin, 1961):

As I walked down Mobile Street, a car full of white men and boys sped past. They yelled obscenities at me. A tangerine flew past my head and broke against a building. The street was loud and raw, with tension as thick as fog. I felt the insane terror of it. (p. 66)

It is this type of racial stress, described here by a White person experiencing it for the first time, that has been a part of the long history and experience of being racialized as Black in America; however, Jim Crow laws also extended into Canada, with the last racially segregated school (Nova Scotia) closing as late as 1983 (James et al., 2010; Maynard, 2017). The singularity of anti-Black sentiment is not just a relic of the past. Statistics show that even in 2018, Black Canadians were more likely than any other racial group in Canada to be the victims of a hate crime (Moreau, 2020; Taylor & Richards, 2019). Racial trauma in North America has required the cultural development of unique coping responses.

All Western nations appear to exhibit some level of anti-Black bias (Faber & Williams, 2019). Although stress reactions can vary from one person to the other and persons of all racial groups experience stress in one form or another, Black people are subject to a unique set of racial stresses that influence the way in which they wield emotion regulation and coping processes. This starts early, because the way in which children are racialized impacts the way they regulate their emotions (Gaylord-Harden & Cunningham, 2009). Studies that have been conducted on this topic explored how Black people specifically regulate their emotions to cope with racist incidents, which is a common occurrence among this population. They also considered how Black people can adapt to and cope with these stressful events.

Clark and colleagues (1999) studied the mechanism through which racism can act as a stressor for African Americans. They suggested a model that integrated biopsychosocial effects of perceived racism on these individuals, noting, however, that the extant research was insufficient. In addition, a selective review of individual-level coping strategies for combating interpersonal racism carried out by Brondolo and colleagues (2009) also emphasized the lack of research focusing on strategies people can use to cope with racism. Thus, more work is needed to understand the current ways that Black people cope with racism and ascertain the most advantageous means of coping. The most advantageous approaches would be defined as “functional” such that they contribute to and maintain well-being.

## Mapping racism-specific coping mechanisms

Faced with a dearth of concrete research, this article provides a comprehensive review of the literature to summarize the research documenting the various ways Black people in racialized societies regulate their emotions and cope when faced with racism. Therefore, we aim to determine how the deployment of these coping mechanisms can vary between men and women and discuss how they may be helpful or harmful. By gathering information from multiple articles from different databases, we compile the available literature including race-specific strategies and identify important gaps to stimulate further progress. We discuss how Black men and women tend to use different strategies to cope with racism and compare this with the experience of coping with physical pain. Finally, we contrast functional and dysfunctional coping responses and highlight the value of empowerment for psychological well-being and social change. We offer these recommendations with the hope that they may inform clinicians and researchers, paving the way toward improved mental health and well-being for all Black people who would be better protected and more empowered to act as agents of social change and address the root of the issues they face—namely racism itself.

## Scoping Review

### Method

An ongoing larger scoping review exploring cross-cultural differences in emotion regulation was initially used to find articles for the current article. The larger scoping review seeks to identify key cultural and emotion-regulation factors that are essential for building a culturally informed model of emotion regulation. However, very few of the 9,257 total articles originally identified for abstract and title screening were related to the topic of Black people’s coping response to racism. Therefore, a wide search for peer-reviewed articles was conducted using the following online databases: PsycInfo, PubMed, MEDLINE, Google Scholar, Microsoft Academic, and Scholars Portal.

Relevant articles were collected by using a combination of the following search terms: “emotion regulation,” “coping strategies,” “racial discrimination,” “racism,” “coping response,” “Black,” and “Black people.”

The initial search resulted in 11,215 articles. The article’s title and abstract were screened to assess for the inclusion criteria. To be included, the article must have been (a) published in English or French, (b) published in a peer-reviewed journal, (c) related to coping or emotion regulation, (d) relevant to discrimination or racism, and (e) included Black people of any ethnicity or nationality. We also manually searched the reference sections of the initially identified articles to find additional relevant articles. After the initial search, 56 articles dated from 1996 to 2021 could be identified.

**Fig. 1.**
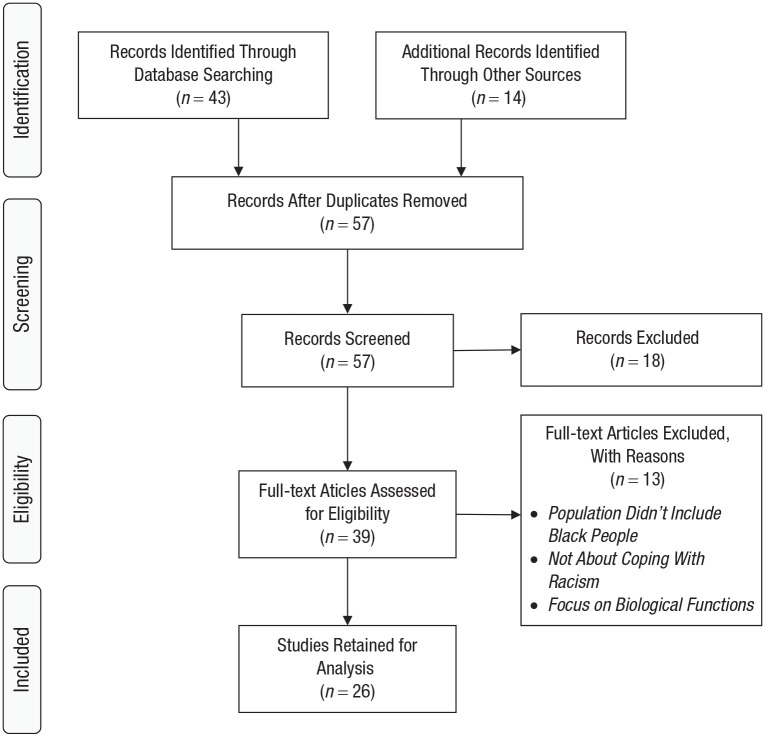
Process of screening and selecting studies for inclusion in this review.

A full text review was subsequently carried out to confirm articles met the inclusion criteria. We did not include articles about racism and psychopathology if they did not also focus on coping. Articles that studied children (under 18 years old) were not included in the project because coping techniques in children would be expected to differ from coping in adults (e.g., S. C. T. Jones et al., 2020). Articles were excluded if the topic was not coping with racism or if they emphasized only biological functions such as blood pressure and heart rate as their measure of coping. Ultimately, 26 articles were included in the current review (for the literature flow diagram, see Fig. 1; Moher et al., 2009).

### Results

The 26 articles selected for review and analysis are listed in Tables 1 and 2 and are ordered by publication year. There were 18 quantitative and eight qualitative studies. Tables 1 and 2 also list the sample size, type of study, and key findings. Quantitative studies were also examined to help ascertain which strategies were connected to positive or helpful outcomes for participants. Notably, the majority of studies were cross-sectional, which limits our ability to draw cause-and-effect conclusions. There were, however, two daily diary studies and two longitudinal survey studies (Hoggard et al., 2012; D. M. Pittman & Kaur, 2018; Sanders Thompson, 2006; Swim et al., 2003), but of these only D. M. Pittman and Kaur (2018) and Sanders Thompson (2006) examined the utility of a coping strategy. Of the qualitative studies, two used focus groups and six used interviews, which is also noted.

**Table 1.** Summary of Literature Review of Black People’s Coping Response to Racism: Quantitative Studies

Reference	Sample	Quantitative methodology	Findings	Outcomes
Plummer & Slane (1996)	*N* = 532 (156 Black Americans)	Experimental design focused on varieties of strategies used: Black vs. White people	Using the Ways of Coping Questionnaire, Black people were found to use more coping strategies when faced with racial stress than White people. Black people engaged in significantly more problem-focused coping (accepting responsibility, confrontive coping, planful problem-solving, and seeking social support) and emotion-focused coping (distancing, escape avoidance, positive reappraisal, and self-control) than White people.	No assessment of how helpful or effective any of the strategies were.
Utsey, Ponterotto, et al. (2000)	*N* = 213 African American college students	Cross-sectional survey study focused on varieties of strategies used: men vs. women	African American women used social support significantly more than African American men. African American women preferred avoidance for individual racism over social support and problem-solving. Seeking social support and cultural racism were significant predictors of race-related stress in African Americans.	Avoidance coping strategies negatively predicted self-esteem and life satisfaction. Problem-solving and social support were generally deemed helpful strategies, but their effectiveness was dependent on self-esteem and life satisfaction.
Swim et al. (2003)	*N* = 51 African American university students	Daily diary study (2 weeks) focused on who reports racism and types of responses: men vs. women	More Black participants tended to respond to everyday racist incidents (42% directly, 21% indirectly, and 33% not at all). Men were more likely to not respond to the incident; women were more likely to discuss the incident with friends (seek social support).	No findings on how helpful or effective strategies were.
Clark (2004)	*N* = 269 Black American university students	Cross-sectional survey study focused on varieties of strategies used: men vs. women	Women were more likely to use emotion-focused and religious coping strategies than men in inter- and intragroup racism. Men were more likely than women to use alcohol to cope in intragroup racist situations.	Black people who perceived less racism scored higher on a measure of self-deception and had increased vulnerability to psychological distress
Lewis-Coles & Constantine (2006)	*N* = 284 African Americans	Cross-sectional survey study focused on Africultrual and religious coping: men vs. women	Higher perceived institutional racism-related stress was linked to a higher use of cognitive/emotional debriefing, spiritual-centered, and collective coping strategies for African American women. Among African American men, higher levels of cultural racism-related stress were associated with a greater use of collective coping strategies.	Higher levels of racism-related stress predicted a higher use of spiritual-centered and collective coping but also higher levels of cognitive/emotional debriefing.
Sanders Thompson (2006)	*N* = 156 (70 African Americans)	Survey data collected at multiple time points after event; focused on varieties of strategies used: Asian vs. Black people vs. White people and men vs. women	African Americans were more likely than Asian and European Americans to seek support and guidance to cope with discrimination. Coping Response Inventory: approach coping (logical analysis, positive reappraisal, seeking guidance and support, and problem-solving) and avoidance coping (cognitive avoidance, acceptance or resignation, seeking alternative rewards, and emotional discharge).	Logical analysis increased reexperiencing symptoms but was associated with less self-blame. Cognitive avoidance decreased reexperiencing symptoms.
Thomas et al. (2008)	*N* = 344 African American women	Cross-sectional survey study focused on varieties of strategies used	Interaction of gendered racism on psychological distress was partly moderated by avoidant coping styles (e.g., cognitive-emotional debriefing). No moderating effects were found from spiritual-centered, collective, or ritual-centered coping strategies.	Avoidant coping styles lessened the negative impact of experiencing psychological distress because of gendered racism.
Joseph & Kuo (2009)	*N* = 190 Black Canadians	Cross-sectional vignette study focused on varieties of strategies used: men vs. women	A combination of general and Africultural coping mechanisms were utilized to manage the stress of racial discrimination at different levels (interpersonal, institutional, and cultural). Five coping strategies were used to respond to racial discrimination: problem-solving coping, cognitive-emotional debriefing, spiritual-centered coping, collective coping, and ritual-centered coping.	No assessment of how helpful or effective any of the strategies were.
Brown et al. (2011)	*N* = 147 African American students	Cross-sectional survey study focused on varieties of strategies used (dispositional vs. situational coping): men vs. women	Religion and venting were the only coping strategies that were used significantly more in racism-related situations. Women’s top three strategies used to cope with racist situations were religion, emotional support, and instrumental support. Men’s top three strategies used to cope with racist situations were acceptance, active strategies, and planning.	No assessment of how helpful or effective any of the strategies were.
C. T. Pittman (2011)	*N* = 366 African Americans	Cross-sectional interview study focused on coping using anger	Anger as a coping mechanism for racial discrimination had a negative impact on African Americans’ general well-being and psychological distress. Men were more likely to use active anger to cope with acute racism.	Active anger led to depressive symptoms.
Hoggard et al. (2012)	*N* = 35 African American women students	Daily diary study (20 days) focused on coping: racial vs. nonracial stressors	More confrontative, ruminative, and avoidance coping were used for racially stressful events. Significantly more ruminative coping were used for racially stressful events.	Racial and nonracial stressors were equally taxing.
Graham et al. (2013)	*N* = 57 African Americans (from sample of 185 Black people)	Cross-sectional survey study focused on mindfulness coping	Mindfulness skills helped buffer the effects of racist experiences on anxiety arousal symptoms. Mindfulness was more useful for acute experiences of anxious arousal from racist experiences.	Mindfulness helped reduce anxiety based on appraisal of past-year events.
Pearson et al. (2014)	*N* = 562 African American university students	Cross-sectional survey study based on retrospective report; focused on types of strategies chosen by participants	The eight coping factors from the Brief-COPE were substance use, religion, humor, self-blame, positive reframing, social support (instrumental/emotional support), active/planning, and disengagement. People higher in neuroticism were more distressed by racism.	Substance use, self-blame, and disengagement were related to neuroticism. For racism, negative psychological reactions may be beneficial in the short run.
Greer et al. (2015)	*N* = 201 African American university students	Cross-sectional survey study focused on disengagement and problem- oriented coping strategies	Four types of coping behaviors for intragroup racial stressors—interconnectedness, problem-oriented coping, disengagement, and spirituality—were moderately correlated. Disengagement strategies were associated with increased stress, whereas problem-oriented coping was correlated with less stress and better academic performance.	Disengagement strategies were associated with increased stress. Problem-oriented coping was correlated with less stress and better academic performance.
Merluzzi et al. (2015)	*N* = 335 (213 African Americans)	Cross-sectional survey study focused on coping strategies (active/agentic vs. disengagement): Black vs. White cancer patients	For African Americans, higher perceived discrimination was linked to higher use of disengagement strategies. African Americans adopted significantly more disengagement strategies compared with White Americans.	Disengagement coping had negative consequences on quality of life. Agentic strategies were feasible (but not always) for African Americans.
Hill & Hoggard (2018)	*N* = 69 African American women	Cross-sectional survey and psychophysiology study focused on two specific coping strategies (rumination and John Henryism)	Through rumination, anticipatory race-related stress was linked to depressive symptoms. Race-related stress was related to a greater tendency to ruminate in African Americans. The relationship between race-related stress and rumination was conditional on degrees of John Henryism (hard-work and determination).	Elements of John Henryism may have protective effects on psychological health but can be detrimental long-term. Rumination is linked to depression.
D. M. Pittman & Kaur (2018)	*N* = 469 African American women	Longitudinal survey study (three waves) focused on substance-abuse coping strategy (alcohol use)	Alcohol consumption among Black college women was substantially related to general life stress and perceived racism. Only an increase in racist experiences enhanced participants’ hazardous drinking. High-risk drinkers reported more distress from life and race-related stressors.	Increased rates of alcohol consumption are subject to general life stress, including racism. Being categorized as a high-risk drinker increased by a ratio of 1.12 for every unit of increased race-based stress.
Riley et al. (2021)	*N* = 419 African American students	Cross-sectional survey study focused on cognitive emotion regulation coping strategy	Higher racial discrimination was related to less use of reappraisal (self-assessment). Experience with racial discrimination was associated with greater community and civic engagement.	Increased discrimination was correlated with greater civic engagement. However, reappraisal was associated with greater civic engagement for females and less for males.

Note: COPE = Coping Orientation to Problems Experienced Inventory.

**Table 2.** Summary of Literature Review of Black People’s Coping Response to Racism: Qualitative Studies

Reference	Sample	Qualitative methodology	Findings
Shorter-Gooden (2004)	*N* = 196 Black women	Interviews	African American women relied on three categories of strategies to deal with the stress of oppression: 1. Internal resources or coping strategies: resting on faith, standing on shoulders, and valuing oneself. 2. External resource: leaning on shoulders. 3. Specific coping strategies for racism and sexism: role flexing (altering), avoiding, and standing up and fighting back.
C. T. Pittman (2010)	*N* = 16 African American faculty members	Face-to face interviews	Female faculty depended on assertive actions to establish their authority for racial stressors in the classroom using five active strategies: 1. Return the focus to a teaching or learning objective. 2. Create and maintain a safe space for White students. 3. Take anticipatory actions. 4. Nonreactively question students’ assumptions. 5. Establish authority through assertive actions.
Holder et al. (2015)	*N* = 10 professional Black Women	Semistructured interviews	Coping strategies to respond to racial microaggressions in the workplace were religion and spirituality, armoring, shifting, social support, sponsorship and mentorship, and self-care.
Hudson et al. (2016)	*N* = 26 African American men	Four focus groups	Respondents used four different categories of behaviors to cope: 1. Health-promoting behavior: physical activity as a coping strategy. 2. Negative health behavior: Men used more negative health behaviors, including smoking, alcohol, and illicit drugs. 3. Social support: reliance on family; time with their children; support groups. 4. Spirituality and religiosity: prayer to relieve stress.
Griffith et al. (2019)	*N* = 12 Black college students	Semistructured interviews	Coping strategies used to respond to race-related stressors were processing the event, selectively seeking social support, working harder and persisting despite discrimination, and educating White peers.
Spates et al. (2019)	*N* = 22 Black women	Semistructured interviews	Coping strategies used to manage racial and gender stressors followed four themes: redefining Black womanhood; using overt and covert forms of resistance; relying on faith, prayer, and the pursuit of balance; and expressing their thoughts and feelings in safe spaces.
E. K. Griffin & Armstead (2020)	*N* = 74 Black people	Eight focus groups	Using a biopsychosocial perspective, seven “pluralities” of both adaptive and maladaptive racial-stress coping were found: avoidance coping, humiliation, overall avoidance of White people, physical reactivity, emotional coping, problem-focused coping, and cultural assimilation.
Volpe et al. (2021)	*N* = 189 Black undergraduate students	Online survey with prompts focusing on which strategies were used	Most (64%) responded to their worst experience of racism with active coping (i.e., social support, the desire to work harder in the face of adversity). Participants used active coping more frequently for institutional-level racism. In addition: 1. Most participants’ responses (85%) were inner-directed. 2. Black women reported more use of active coping strategies than Black men, with 64% of Black women utilizing social support. 3. In response to their worst experience of racism, 35.4% reported using a passive coping technique, such as accepting, avoiding, rejecting, minimizing, and/or brooding, 4. Fifteen percent of participants reported outer-directed coping, which tended to center on teaching others and confronting racist public displays with offenders. 5. Black men tend to use self-control and moderate outward behavioral presentation. 6. Participants mentioned creating poetry (artistic endeavor) to cope with racism.

These studies evaluated the common coping strategies used by Black Americans and Canadians, often with a specific focus on how they impact mental-health variables, such as depression and anxiety symptoms (Graham et al., 2013). None of the studies differentiated between sex and gender, and few differentiated between race and ethnicity. Most of them did not differentiate between Black people and African Americans; exceptions are Graham et al. (2013), Griffith et al. (2019), Spates et al. (2019), and Volpe et al. (2021). However, surprisingly, none reported results separately by ethnic group or made subgroup comparisons. Of the 26 articles, 25 were from U.S. samples and one was from a Canadian sample; no other countries were represented. Given the predominance of American literature, the remaining findings will best apply to Black Americans.

The two most frequently mentioned coping mechanisms were religion and social support; however, there was variability in regard to the other coping mechanisms used and studied. Strategies that were presented for Black Americans and Canadians can be found in Table 3, which presents a complete overview of all the coping strategies mentioned in the articles reviewed.

**Table 3.** List of Coping Strategies Studied

Category	Assessment
Avoidance (3) Disengagement, not responding (2) Distancing, escape avoidance (4) Cognitive emotional debriefing (avoidance and denial) (3) Self-blame/accepting responsibility (2) Ritual-centered coping (1) Assimilation (1) Shifting (1) Substance use (2) Smoking (1) Drugs (licit and illicit) (1) Alcohol (1) *Food*	Dysfunctional
Cognitive strategies Processing the event (1) Positive reframing/reappraisal (2) Acceptance (1) Mindfulness, meditation (2) Meaning making (1) Planning (2) Physical strategies Physical activity (2) John Henryism, working harder (4) Resisting retaliation (impulse/self-control) (2)	Ambiguous
Social support (10) (friends and family) Collective coping, support groups (1) Instrumental support (2) Venting (1) Humor (1) *Therapy* (1) Direct strategies (3) Problem-solving (3) Covert resistance (1) Agentic strategies (1) Speaking out (1) and confrontation (2) Active anger (1) Identity affirmation Positive self-statements (2) Africultural coping (2) Spirituality/religion (6) Art (1) Activism (1) Public resistance Educating others (2) Community/civic involvement (1)	Functional

Note: The coping strategies found in the 26 review articles were categorized under the eight general headings listed in this table. Numbers in parentheses represent the number of articles that mentioned the heading or the specific subcategory. Some articles mentioned several strategies. Italicized coping strategies have been indicated in the literature but are not represented in the cited articles.

**Fig. 2.**
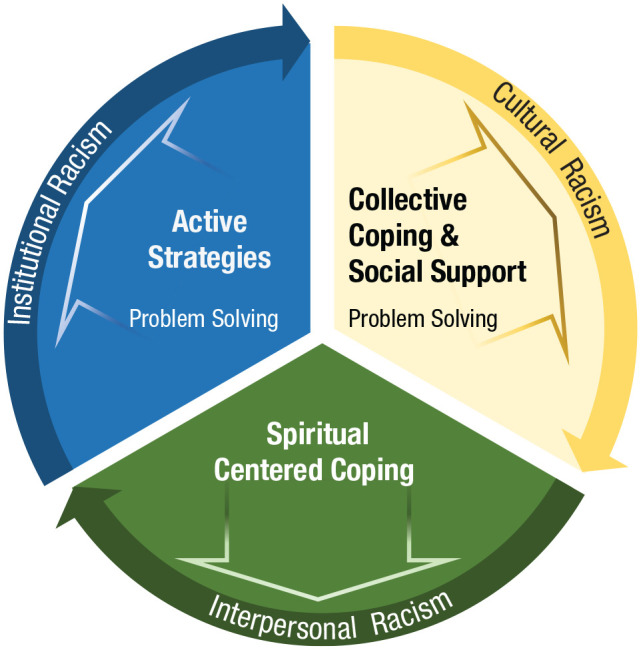
Coping-strategies model. Types of racism are depicted as a linked circle. Categories of strategies used by Black individuals are wielded preferentially in a specific way to cope with each of the three types of racism.

We found that the type of racist experience determined to a great extent which type of strategy people reported using. We identified three distinct levels of racism in the studies: cultural (i.e., assertion of Eurocentric Western values and practices resulting in the exclusion or denigration of other histories and traditions), interpersonal (i.e., biases that occur when an individual’s racialized beliefs affect their interactions with people of color), and institutional (i.e., differential access to goods, services, and opportunities based on perceived racial identity; see Fig. 2; Haeny et al., 2021; J. M. Jones, 1999).

Of the 26 articles, four highlighted differences in the coping response to all or one of the types of racism listed. People reported using a combination of separate strategies to combat institutional racism, including active coping (Volpe et al., 2021) and problem-solving strategies (Joseph & Kuo, 2009). If faced with interpersonal racism, individuals emphasized spirituality-based strategies. In contrast, in the face of cultural racism, individuals chose collective coping, social support, and problem-solving (Utsey, Ponterotto, et al., 2000).

Although the cited articles show how universal many of these coping mechanisms are for Black Americans, there has been no general consensus on their efficacy as tools to combat racial stress and trauma. Observing how different forms of coping are habitually used for specific types of stressors is a useful insight for designing experiments and interventions that can inform researchers and clinicians as to which of these types of responses are most efficacious in which situations.

### Gender comparisons

The majority of studies found that Black people used a range of strategies in response to racism (Brown et al., 2011; Clark, 2004; E. K. Griffin & Armstead, 2020; Hoggard et al., 2012; Hudson et al., 2016; Joseph & Kuo, 2009; Lewis-Coles & Constantine, 2006; Pearson et al., 2014; C. T. Pittman, 2010; Shorter-Gooden, 2004; Spates et al., 2019; Utsey, Ponterotto, et al., 2000; Volpe et al., 2021). Within this variety exist several recurring coping mechanisms used widely by Black Americans regardless of gender or other factors. Multiple articles show they use religion, social support, and problem-focused coping to respond to racist experiences. Social-support mechanisms were outlined in seven publications (Griffith et al., 2019; Joseph & Kuo, 2009; Lewis-Coles & Constantine, 2006; Pearson et al., 2014; Sanders Thompson, 2006; Utsey, Ponterotto, et al., 2000; Volpe et al., 2021), five studies included religion (Brown et al., 2011; Greer et al., 2015; Joseph & Kuo, 2009; Lewis-Coles & Constantine, 2006; Pearson et al., 2014), and four included problem-focused coping (Greer et al., 2015; E. K. Griffin & Armstead, 2020; Joseph & Kuo, 2009; Plummer & Slane, 1996). In addition, the authors had similar interpretations of the usefulness of these strategies. Religion, for example, referred to attending church, prayer, and spirituality. Problem-focused coping encompassed active efforts made by an individual to directly confront the stressor to eliminate, modify, or reduce it, whereas social support described attending support groups such as Alcoholics Anonymous or talking with friends or family (Schoenmakers et al., 2015).

#### Black women

Many of the studies reviewed here focused specifically on coping mechanisms used more frequently by Black women than by men. Seeking social support, for example, was found to be incredibly important for Black women in eight independent studies (Brown et al., 2011; Holder et al., 2015; Lewis-Coles & Constantine, 2006; Shorter-Gooden, 2004; Spates et al., 2019; Swim et al., 2003; Utsey, Ponterotto, et al., 2000; Volpe et al., 2021) and was described in one study as a “buffer” from “the sting of oppression” and a reminder that they were “never alone when adversity arises” (Shorter-Gooden, 2004, p. 417). Ultimately, Black women used social support to validate their difficult experiences and felt less alone in their struggles.

Religion and spirituality is another coping strategy that follows a similar pattern. It was categorized as significantly more popular for Black women in six different studies (Brown et al., 2011; Clark, 2004; Holder et al., 2015; Lewis-Coles & Constantine, 2006; Shorter-Gooden, 2004; Spates et al., 2019). Participants in Spates et al.’s (2019) study categorized religion as a way to stay optimistic and joyful in spite of the hardships they face.

Finally, other types of strategies that were observed in Black women were “overt strategies” (or agentic strategies) that were mentioned in three studies (C. T. Pittman, 2010; Shorter-Gooden, 2004; Spates et al., 2019). This term encompasses observable responses such as confronting or speaking out (McCarty et al., 1999). In C. T. Pittman (2010), female faculty used assertive actions in the face of classroom racial stressors and to reestablish their authority. One of the Black female faculty members described how she spoke up for herself after a White student threw paper at her (C. T. Pittman, 2010). Likewise, Black women in Spates and colleagues’ (2019) study used overt strategies such as calling out discriminatory behavior. Furthermore, a participant in Shorter-Gooden’s (2004) study used active strategies to combat racism by filing a report against an officer after experiencing police abuse. Thus, as exemplified by numerous articles in this review, there are many ways overt strategies can be enacted.

In addition, “covert strategies,” which are intrapersonal actions not readily observed by others, were also observed (Aldao & Dixon-Gordon, 2014). Some Black women described trying to blend in and not stand out to avoid racism by assimilating, achieving integration through behavioral and attitude modification (Spates et al., 2019). Black American women will often adjust their behaviors and roles to reduce barriers (Hall et al., 2012). Avoidance strategies were used by Black women in four studies (Lewis-Coles & Constantine, 2006; Shorter-Gooden, 2004; Thomas et al., 2008; Utsey, Ponterotto, et al., 2000). Avoidance coping comprises avoidance of the stressors instead of becoming actively involved with them and includes minimizing or denying noxious behaviors (Holahan et al., 2005). The cognitive-emotional debriefing coping style (Utsey, Adams, & Bolden, 2000) was identified in three studies. This strategy entails forgetting about the situation, minimizing the negativity associated with the situation, and/or taking part in distracting activities (Joseph & Kuo, 2009; Lewis-Coles & Constantine, 2006; Thomas et al., 2008). Thomas et al. (2008) found that when more gendered racism is experienced by a Black woman, it will lead to more distress and more engagement in cognitive-emotional debriefing. Despite the misleading name ascribed to this strategy, it would be considered an avoidant approach (e.g., analogous to the concept of experiential avoidance).

Responses to the three different levels of racism identified in the previous section also differ by gender (Fig. 2). For interpersonal racism, African American women preferred avoidance strategies (Utsey, Ponterotto, et al., 2000), whereas they responded with spiritual-centered strategies, cognitive-emotional debriefing, and collective coping strategies when dealing with institutional racism (Lewis-Coles & Constantine, 2006).

In sum, Black women use a variety of strategies, both overt and covert, to cope with racism, the most common being social support and faith-based strategies. Although used more heavily by Black women, these two coping mechanisms are very common among the general Black populous regardless of sex.

#### Black men

In regard to the different levels of racism (Fig. 2), African American men used collective coping strategies that included social support from the community, family, and friends when faced with cultural racism (Lewis-Coles & Constantine, 2006). Although this trend was identifiable, other trends relating to the coping styles of men were less clear-cut.

In contrast to literature detailing the coping mechanisms for Black women, we did not find uniform strategies among Black men. Strategies included seeking social support (Hudson et al., 2016), active anger (C. T. Pittman, 2011), substance use (Clark, 2004; Hudson et al., 2016), planning (Brown et al., 2011), religion (Hudson et al., 2016; Lewis-Coles & Constantine, 2006), not responding (Swim et al., 2003), various active strategies (Brown et al., 2011), and acceptance (Brown et al., 2011). On the basis of the literature to date, it is difficult to say with certainty which of the strategies used by Black men to cope with racist incidents are the most common or effective.

One distinct difference in coping strategies between Black men and women was that passive strategies such as ignoring (Table 4) were used more frequently by men than by women. These types of strategies are highlighted in Table 4, which compares coping strategies by gender and physical versus emotional pain caused by racism. The gender difference could be attributed to the ways in which our racialized society punishes Black men much more harshly for using strategies in which the agency is externally visible (e.g., Williams, 2020). Thus, speaking out and confrontation were primarily used as a coping strategy by Black women. The heightened use of physical activity as a coping mechanism by Black men compared with women can be seen as compensation in taking back their agency in a socially acceptable (but very circumscribed), external way. It is one of the only avenues that remains open to them because of the intensive scrutiny and surveillance many of their actions are subjected to.

**Table 4.** Comparison of Coping Strategies

				Coping Categorization		
Coping strategies for physical pain (Meints et al., 2016)	Coping strategies for emotional pain and stress of racism	Men	Women	Cognitive/behavioral	Active/ passive	Problem-/ emotion- focused
Relaxation				Behavioral	Active	Problem
Catastrophizing				Behavioral	Active	Emotional
Hoping/praying	Identity affirmation (11)	Religion, emotion-focused coping	Religion, emotion-focused coping	Cognitive	Active	Emotional
Seeking social support	Social support (16)	Problem-focused social support	Problem-focused social support	Behavioral	Active	Emotional
Ignoring pain sensations	Avoidance (9)	Avoidance, nonresponse	Avoidance, assimilation	Behavioral	Passive	Emotional
Diverting attention	Cognitive emotional debriefing/ritual (4)	Self-blame, denial	Self-blame, denial	Cognitive	Active	Problem
Coping self-statements	Acceptance (1)	Acceptance		Cognitive	Active	Problem
Reinterpreting pain	Positive reframing/reappraisal (2)	Reappraisal	Reappraisal	Behavioral	Active	Problem
Increasing behavioral activity	Agentic strategies (8); activism (4)	Active anger, self-control	Speaking out, confrontation, civic engagement	Behavioral	Active	Problem
Exercising and stretching	Physical activity (2)	Physical activity		Behavioral	Active	Problem
Task persistence	John Henryism (2)	Working harder		Behavioral	Active	Problem
Guarding	Processing/mindfulness/meditation (6)	Planning	Rumination	Cognitive	Active	Problem
Not listed	Substance use (5)	Substance use	Substance use	Behavioral	Active	Problem

Note: This table compares coping strategies by sex and physical versus emotional pain (to the stress of racism). Side-by-side comparisons allow a birds-eye view of physical-pain strategies that are not used in coping strategies to racism versus those preferred and often used for both types of pain. Numbers in parentheses represent the number of times the category or substrategy was mentioned in Table 3; more than one coping strategy was mentioned in most articles.

## Comparing Coping With Racism and Physical Pain

### Pain and coping

Coping with racism is psychologically taxing and even painful. Although not identical, there are commonalities between how the brain processes physical versus emotional pain (Sturgeon & Zautra, 2016). Functional MRI (fMRI) studies have shown that physical pain and social rejection activate common brain regions (Eisenberger, 2012; Novembre et al., 2014). Furthermore, coping mechanisms defined as “active methods” can be seen successfully regulating pain in real time through fMRI studies (Emmert et al., 2017). A meta-analysis also demonstrated that cognitive and meditative therapies can alter the functioning of brain regions and reduce the affective experience of pain (Nascimento et al., 2018). These examples demonstrate a degree of usefulness in using active coping strategies to reduce and regulate pain. There is also an observed commonality between the coping strategies as reactions to both physical and mental pain, as reviewed by Sturgeon and Zautra (2016). Armed with this knowledge, it is well within the realm of possibility to impute common resiliency mechanisms used by African Americans for coping with physical and emotional pain alike (Gorczyca et al., 2013; Sturgeon & Zautra, 2016).

### Racial differences in coping with pain

For this reason, it may be instructive to reassess the literature examining the differences between how White and Black Americans cope with physical pain and contrast this with data generated here in comparison. The first holistic publication to examine and quantify the relationship between race and the use of pain-coping strategies, a meta-analysis, found that for physical pain, Black individuals tend to use more types of coping strategies more frequently than White individuals (Meints et al., 2016). Specifically, Black individuals utilize five separate strategies—praying and hoping, diverting attention, catastrophizing, and reinterpreting pain sensations—all with more frequency than White individuals. White people, in most cases, made use of a single strategy (task persistence) and relied on this strategy more frequently than African Americans. The magnitude of the differences observed between racial groups was larger for passive versus active strategies, emotion-focused versus problem-focused strategies, and cognitive versus behavioral strategies (Meints et al., 2016).

**Fig. 3.**
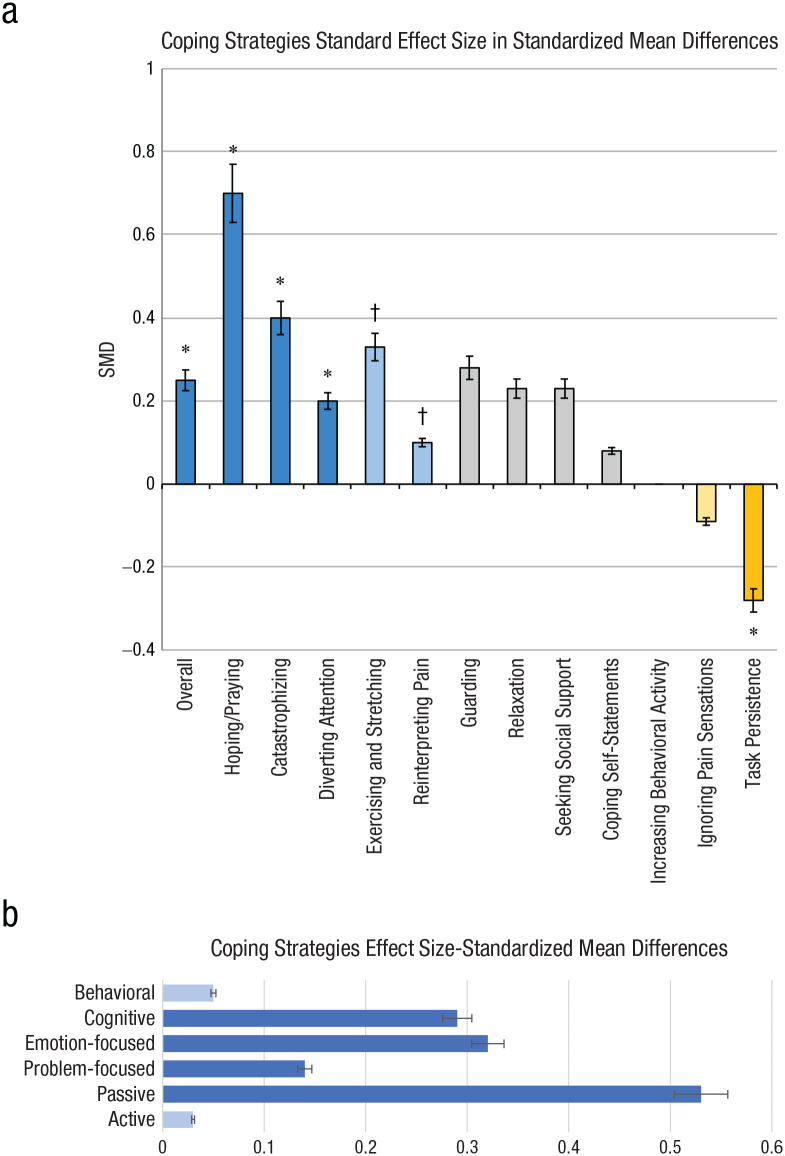
Coping strategies for pain and effect sizes. As shown by the reconceptualized graph of data from Meints et al.’s (2016) meta-analysis of the effect sizes for coping strategies as utilized differentially between Black and White Americans (a), Black people (blue bars) used more types of coping strategies than White people (orange bars), and although there were some commonalities, each race largely had specific coping-strategy preferences. The asterisk indicates significance. Black people (blue bars) used cognitive, emotion-focused, problem-focused, and passive strategies more than White people (b). Mean sizes below 0.1 (light blue bars) were not significant. As one of the only significant empirical meta-analyses focused on responses of Black individuals, there is a recognizable overlap between Black-specific coping strategies for physical pain and emotional responses, providing some indication for the validity of current findings, although it is not divided by gender.

A reconceptualized graph of data from Meints and colleagues (2016) focusing on effect sizes for pain-coping strategies used by Black Americans contrasted with White Americans outlines the wider array of strategies used (Fig. 3). The coping mechanisms used by Black participants in response to racism were somewhat similar to but also notably different from those utilized by Black participants in response to physical pain.

Meints and colleagues (2016) identified 12 specific types of coping strategies for physical pain, which were subcategories of six broader categorizations (Table 4, column 1). When grouping the specific strategies into these categories (Table 4, column 2), the differences and similarities become clearer. Although Black individuals use catastrophizing or relaxing as coping strategies for physical pain, these strategies do not seem to be used at all to cope with racism. After creating this comparison, we noted an absence of any relaxation coping strategies in the face of the stress of racism, which is alarming. In therapeutic practice, it can be a hurdle to convince Black patients suffering from burnout to take even one day off, which may be due to the stigma of Western myths surrounding the work ethic of people racialized as Black (e.g., Quaye et al., 2019). The epidemic underutilization by Black people of self-care is in itself worthy of further research and an indication that more self-care may be warranted.

Black Americans are, however, using some of the same strategies used for coping with physical pain (Table 4) to cope with emotional pain, although these strategies differ somewhat by gender. When visualized in this way, a few interesting trends are immediately visible. First, religion is a widely utilized coping strategy for both physical and emotional pain for both genders. The use of social support among Black participants is also a common coping strategy for physical pain and for pain caused by racism. Ignoring pain is a strategy that is rarely used by Black people when the pain is physical. However, avoidance strategies, including ignoring, are used by both genders in response to racism. Physical activity, in contrast, is a coping strategy used for both physical and emotional stress, but it was only noted for Black men when coping with racism. Substance use, although not included as a coping category in Meints et al. (2016), has been documented in many other studies as a means of coping with physical and emotional pain (Garland et al., 2013; Heggeness et al., 2019; Novak et al., 2009).

Interestingly, women and men sometimes had opposite outcomes for the same strategies for coping with racism (Table 4, row 9); although both strategies are behavioral, active, and problem-focused, women tend to utilize activism as a response to racism, whereas Black men do not cope in this way but rather tend to choose to respond with self-control (Riley et al., 2021; Volpe et al., 2021). This may be indicative (again) of the very different societal punishment relegated specifically to Black men.

This kind of comparative mapping allows us to see coping trends from a bird’s-eye perspective to make useful inferences. Interestingly, in general, Black people rarely use passive coping strategies for emotional pain, although they do somewhat for physical pain. Passive coping strategies for racism (Table 4) include only nine examples, in contrast to a full 54 active strategies. The idea of having agency would therefore seem to be a common thread in coping strategies for racism; the ability to be able to do something (anything) links the responses of Black people and is notable in this context, although not all active strategies are positive (i.e., cognitive-emotional debriefing). Certain coping strategies that incorporate agency are also called “agentic” (Table 3) in the literature (Merluzzi et al., 2015). Here, it has been specifically noted that agentic coping strategies are effective but not always feasible for Black people because of racism backlash. In a trauma such as racism that is caused by the lack of agency, the determination of an individual to take up agency in any way no matter how small to reclaim a sense of autonomy is therapeutic. The frequent use of active coping identified here testifies to the potential usefulness of such strategies for future holistic-therapy approaches developed to mitigate trauma-induced racism, and such approaches should include teachings on how to find positive agency.

## Discussion

Men and women primarily use the same categories of coping strategies (identity affirmation and social support); any differences appear to be shaped by cultural forces and social latitude (agentic, activism, and physical activity), with activism being more dangerous for men than women and physical activity being more culturally accepted for men than women. The interesting commonality between coping strategies used by Black people for both pain and racism are seen in that the most utilized strategies for pain (hoping/praying) and for racism (identity affirmation) are connected by a spiritual thread, and again, these strategies seem to have been shaped by similar cultural forces; the spirituality developed by the African diaspora in America developed in part to cope with centuries of brutal pressures by a hateful society. Further research in this area is clearly needed to have a more in-depth understanding of the relative efficacy of these common strategies.

### Functional versus dysfunctional coping methods

It should be relatively clear that some coping methods for racism are more functional than others (Table 3). How functionality is defined will determine whether the goal is to stop acts of racism from occurring or to reduce the emotional distress caused by racist acts in the short term. The former may result in increased stress in the short term but could offer greater dividends in the long term (Jacob et al., 2021). Although not all racism can be prevented, individuals can reduce racism they experience through deliberate actions, such as changing their environment, confronting offenders when safe to do so, and reporting more dangerous perpetrators. Actions that are instead focused on changing one’s emotional or mental state may have a more immediate impact in terms of reducing distress, but actions that have greater utility in putting a stop to racism may end up being a more useful method of coping and facilitate a reduction in racism in the person’s overall social environment. That said, it is important to keep in mind that, at an individual level, even the most dysfunctional approaches have an important purpose for the person making use of it, and more beneficial approaches may not be available or apparent to them.

We expound on two distinct categories of coping strategies, emotion-focused coping (Table 4, rows 3–5) and problem-focused coping (Table 4, rows 6–13), and each of these has different ways of being effective. Emotion-focused coping involves all efforts made to decrease the emotional impacts of a stressful experience (Schoenmakers et al., 2015). The following strategies are included in this category: mindfulness, positive reframing, venting, acceptance, and processing the event. Problem-focused coping involves all active efforts made by an individual to handle stressful experiences by modifying or eliminating the stressor. Speaking out and confrontation are examples of problem-focused coping. This type of coping style can be helpful or harmful. A full list can be found in Table 4.

#### Dysfunctional coping

Many frequently used coping strategies such as problem- and emotion-based coping can have detrimental effects on an individual’s psychosocial function. In turn, this can give rise to more dysfunction in the individual. One example of a harmful coping mechanism often used by Black Americans is John Henryism, a work ethic famously named after the fable of the Black man that worked himself to death competing against a steam engine. This coping style is particularly prevalent in Black middle-class families who believe that enough hard work will eventually lead to them being successful and recognized as equal to their White counterparts (Bennett et al., 2004). Some articles have identified this strategy as being positive in the short term because it can promote hard work and minimize conflict (Hoggard et al., 2012). However, in the long term, it is found to cause or accelerate debilitating physical ailments (Felix et al., 2019; S. Jones et al., 2019; Volpe et al., 2018). Other types of dysfunctional coping mechanisms include rumination and disengagement, which can result in or be caused by depression (Koppe & Rothermund, 2017; Nolen-Hoeksema, 2000); avoidance (including cognitive-emotional debriefing), which increases anxiety in the long term (Hofmann & Hay, 2018); unrealistic positive reframing or self-blame, which can intensify denial and may result in internalized racism and depression (Zahn et al., 2015); and problematic substance use, which can bring about negative outcomes such as dependence and health problems (D. M. Pittman & Kaur, 2018).

#### Ambiguous coping

The manner in which coping strategies are used can determine whether they are helpful or harmful to individuals. Humor is a strategy used by many to cope with stressful situations. Research has demonstrated that positive (good-natured) humor can be a useful emotion-regulation strategy for negative emotions because it helps enable reappraisal of the situation in a less harmful way (Samson & Gross, 2012), whereas negative (mean-spirited) humor creates emotional distance from negative experiences more akin to avoidance strategies and engenders an overall hostile attitude.

Discerning when to avoid escalation by not responding is a critical skill for Black Americans and can also be an essential survival mechanism. An individual can evade negative or even dangerous confrontations that often can be deeply triggering or intensely traumatizing (e.g., Smith et al., 2007). This type of avoidance can ensure personal safety both on a physical and emotional level. However, not responding also causes several long-term psychological issues. For one, this coping strategy can lead to the internalization of racial trauma, which in turn can cause internalized self-hatred, repressed anger, and depression, which can later surface in self-destructive or unhealthy manifestations (e.g., Greer et al., 2015). It also depletes a person’s sense of autonomy and agency.

Confrontation coping has shown to be useful in creating good outcomes such as psychological forgiveness as well as decreased arousal and positive emotions (Hershcovis et al., 2018; Lohr et al., 2007). Likewise, speaking out in other forms can be equally beneficial because it facilitates agency and allows the individual to overcome feelings of powerlessness (Sue et al., 2019). In cases in which confrontation results in heightened risks of anger, persecution, or retaliation from others, speaking out can be dangerous and should be carefully considered (Bushman, 2002; Hershcovis et al., 2018).

Although anger can be a motivator for positive changes and is a normal response for those who feel wronged, active anger can be destructive in several ways. It can lead to impulsive actions or words that are emotionally abusive or violent, and suppressing one’s anger can lead to symptoms of depression (C. T. Pittman, 2011). The consequences of showing anger are higher for Black people than others (Hutchings & Haddock, 2008) because it can result in the involvement of law enforcement. Therefore, Black Americans must exercise more self-control and avoid public displays of anger.

Last, acceptance and mindfulness are two strategies that can have positive impacts for coping because they provide mechanisms for accepting the emotions caused by racist events and decrease anxiety (Brockman et al., 2017; Graham et al., 2013; Hwang & Chan, 2019); however, they can potentially become a net negative if they bring about the acceptance of recurrent racist mistreatment and allow for constant exposure to racism that leads to even greater racial stress or trauma (Sobczak & West, 2013; Williams, 2020). They are also not an effective means of stopping future occurrences of racism.

#### Functional coping

Numerous coping strategies mentioned in this article have been identified as helpful for coping with general life stressors, in particular physical pain (Meints et al., 2016), which provides some evidence that they may be beneficial for the emotional pain of coping with racism as well. All of the helpful strategies are active in nature and can be explored on a spectrum from least active to more active coping strategies.

Planning is the least active coping strategy as a response to racism. This strategy involves preparing for the emotional impacts of racism by actively examining the manner in which to cope with a future event (Brown et al., 2011). Although it is a thought exercise, the focus of this strategy is on the future and not the past or the present. Studies have shown that people of color may begin coping before experiencing a racial event in anticipation of that situation to reduce the effects of this stressor (DeLapp & Williams, 2021). More research should be conducted to address the paucity of literature that exists in relation to the use of prestressor coping in response to racial discrimination.

Moving down the spectrum, the act of venting involves speaking to yourself or another person emotionally about a past experience, although venting can also be journaled. This strategy offers the possibility of processing racial incidents in a concrete way as well as externalizes the event (Brown et al., 2011). Venting can be carried out in the presence of another person; however, it essentially describes a process of speaking out critically about a past event.

Social support is one of the most beneficial and effective coping mechanisms used to counter racial trauma (Driscoll et al., 2015). Social-safety networks and communities give individuals a way to express themselves, provide self-care, obtain feedback, assume agency, and establish resilience. Further, social networks contribute to external positive affirmation. Positive affirmation from a source external to oneself is deeply therapeutic, which is why it is so effective (Stock et al., 2018). Other forms of coping such as the aforementioned venting prove more successful when an individual has strong social connections to fall back on (Seawell et al., 2014).

Racism results in an emotional insult to one’s identity. Therefore, forms of increasingly active and targeted coping mechanisms such as Africultural coping and religious practice that minister to and offer antidotes specifically for injury to the identity represent higher order coping strategies. We call these *identity-affirming* coping strategies (e.g., Anderson et al., 2019). Africultural coping examines a racist event through the lens of identity and may use community and social support in a safe and uplifting manner (Stock et al., 2018; Utsey, Adams, & Bolden, 2000). The result is essentially an act of reclaiming the value, beauty, and purpose of an individual from the attempt of racism to negate one’s identity. It is different, however, from other social-support networks such as religion, because the whole of one’s identity cannot mentally healthfully be defined through ethnonationalism, which is central to the concept of Africultural coping.

The most commonly used coping strategy is religious practice. Religion is among the most active strategies and is frequently utilized by women for several reasons. Religion can offer an established and extensive positive viewpoint on self-identity and provide meaning as to a person’s role in the world. For many Christian Black Americans, the broader perspective has roots in the undeniable intrinsic worth and beauty of the Black individual and the specificity of purpose for which they have been created (Ellison, 1993). This value lies outside of Western value systems and therefore even if disputed by racist thought supersedes its effects. As a coping strategy, through religious inquiry, Black people remind themselves that their mental and physical attributes serve a positive preordained purpose. This understanding of one’s higher purpose can replace the distorted image of oneself that is conjured by racism and likewise thwarts anti-Black racist indoctrination.

Positive affirmation of identity is, through religious inquiry, also linked with the concept of transformative meaning. *Transformative meaning* is a type of positive reframing that can be effective when used as a strategy to reframe suffering, as that occurring from a racist event. It is the well-founded concept that negative, and even events that are traumatic, are able to provide character-redeeming experience if they can be seen as a learning experience on a life-long journey (Miller et al., 2020; Peterson et al., 2008). This thinking, if grounded in the framework of a belief in a benevolent creator with a positive intent and purposeful identity, can also be effective.

Finding a positive purpose and reclaiming identity through transformative meaning is a powerful coping mechanism (Stock et al., 2018). Although the study of coping with racism is modern, these techniques have been in use since antiquity. In an example of how historical figures have used coping mechanisms to deal with racism, consider a famous poem created by the great Black Renaissance professor and linguist Juan Latino (1518–1594), the son of the Count of Cabra in Cordoba and an enslaved Ethiopian woman. He had renamed himself after winning his freedom, and he was active in noble society around the time Spain was on the cusp of a racial holy war—the extermination of Jews and people of color. In the following verse, he reflects on racial relations, among other contemporary events, writing:

Pious kings often keep wonderful things

 at court, so they can show them off to other kings.

Generations of rulers, the Power of Rome itself

 might rightly envy you, [newborn king] Philip, for having a Black poet. (lines 35–38)

Here, after taking up agency by renaming himself, he uses humor, venting, and a transformative cognitive process with self-affirmation of his identity to cope with the nascent anti-Black racism of his time. Both the variety of coping mechanisms and the variety within categories of coping testify to the creative resiliency of humans as demonstrated by the prose that suffering has left behind. The preference by Black individuals for identity-affirming coping strategies that allow individuals to reclaim their identity and reaffirm their self-worth and dignity can explain the frequent use of such strategies. These higher order identity-affirming methods may provide better results as effective coping strategies. Future studies should be designed to confirm these observations.

### Eliminating racism and empowerment

Although helpful in the short term and on a personal level, many of these strategies may not be effective in alleviating the fundamental cause of the pain being experienced or in making meaningful reductions in the lived experience of racism. Genuine social change requires a commitment to strategies that reduce racism everywhere. An understanding of the history, source, and nature of racism is a necessary prerequisite for remedying the stress experienced by Black individuals caused by racism, because it can be difficult to come up with solutions when the core nature of the issue is incompletely grasped. Black people will be best prepared to cope with racism when they understand how racism operates, feel secure in their identity, and are well equipped to address racism as it arises in the moment (Jacob et al., 2021). In this way forceful personal agency in the face of racist actions can serve a therapeutic purpose. It is empowering when an individual can choose how to respond in the moment to a racist confrontation, and it imparts an added measure of control over the discriminatory experience (e.g., Sue et al., 2019).

Problem-focused coping, including direct strategies such as active confrontation, agentic coping, or resolution of a specific racism event, are strategies that can be successful as coping mechanisms; however, they must be carefully navigated. The possibility of asymmetrical backlash from powerful individuals is a risk. If possible, active-confrontation situations should be designed with community (support networks) and not individual involvement in mind. Utilizing these types of coping activities such as activism, that address structural racism issues, is a more beneficial avenue. With such coping activities it is possible to address the source of racism; this can provide a satisfying and therapeutic coping experience that will reap long-term benefits not just for the individual involved but for society at large (e.g., Carlson et al., 2018). Structural racism, as embodied in housing, employment, and education, represent the major community burden of racism, and proper targets therefore for change, because their resolution can contribute to a more wide-ranging alleviation of racism. Activities as coping strategies that specifically address these community burdens can be therapeutic in ways that lift up both the individual and the community and therefore create a positive feedback loop and make the community itself more resilient against racist threats. Additionally targeted and thoughtful community involvement in itself increases the number and quality of outlets for social support. Such communal activities can be as varied as the creation of a community garden, after-school tutoring, removal of polluting/environmental eyesores, cataloguing Black-owned business, classes on homeownership, or how to build a resume (Haldane et al., 2019; Lam et al., 2016).

One helping behavior that can be an effective coping method is educating others about the way racism operates (Table 3). This behavior also provides a virtuous circle by decreasing the general level of racism in the community. Awareness of racism helps the community understand how they themselves commit acts and contribute to racist systems, and in turn this awareness allows them to more easily make antiracist choices.

Notably absent from coping strategies were self-focused self-care approaches—behaviors strictly for personal well-being. These would include things such as fitness, relaxation, enjoyable personal pursuits, time off work, shopping, massage, individual psychotherapy, aromatherapy, listening to music, and turning off the phone and unplugging from stressful social media (e.g., Hansson et al., 2005; Quaye et al., 2019). Only one study in our review mentioned exercise (Hudson et al., 2016). Many authors have noted the benefits of self-care in terms of recovering from racial stress and trauma (Bryant-Davis & Ocampo, 2006; Quaye et al., 2019). It could be that Black people feel they cannot relax or pursue pleasurable activity lest they be judged in accordance with negative stereotypes as being lazy. Taking time for one’s own personal wants and needs could be viewed as an act of empowerment. This is clearly an area in need of future study.

Empowerment is essential to enable racialized people to move toward eliminating racism, which is what is ultimately needed. Positive social change will occur as racism becomes increasingly unacceptable and society becomes more equitable. Making value-based contributions to anti-racist and social-justice causes that work to dismantle racism can be a coping act of agency and self-affirmation (Hope et al., 2018; Jacob et al., 2021).

### Clinical implications

For clinicians seeking ways to support Black clients with racial trauma, the successful coping strategies enumerated here can serve as model starting points and should provide clients with greater agency and better outcomes (Heard-Garris et al., 2021; Hope et al., 2018) than the use of an ambiguous strategy. Therapy should be palpable positive affirmation; clients should feel validated and empowered. If they are religious, finding purpose in their experience even if it was negative can have a positive therapeutic effect. Helping clients find a coping strategy that affirms their intrinsic worth and beauty can also be profoundly therapeutic. If clients do not have affirmative social-support networks, or have dysfunctional social support, helping them find positively affirming support can be highly beneficial. Encouraging clients to create and make art, music, or prose out of their racist experience through positive reframing can be a transformative and proactive coping mechanism (Miller et al., 2020; Stuckey & Nobel, 2010). Certain forms of activism furthermore seem to have specific mental-health benefits (Heard-Garris et al., 2021; Montagno & Garrett-Walker, 2022; Riley et al., 2021). Ensuring that the coping mechanism chosen allows clients to reclaim their identity and dignity is essential. It is important to keep in mind that activism comes in many forms and may or may not involve formal protests or a Black Lives Matter event (E. K. Griffin & Armstead, 2020). Black clients can look for opportunities to promote antiracist change in their personal environments as well (work, school, community) through any number of prosocial means. For a cognitive-behavioral approach to helping clients with racial stress and trauma, see Williams et al. (in press).

### Limitations and future directions

After reviewing the literature, it is clear that much remains to be learned about the role of emotion regulation and coping as strategies for individuals navigating racism. As evidenced by the summary tables, nearly all of the articles reviewed here come from U.S. examples. The exception is Joseph and Kuo’s (2009) study that was done in Canada and focused on Black Canadians. It was rare to identify studies that addressed racialized Black populations other than those in the United States, which may limit the generalizability of the findings.

Racial discrimination based on skin shade is prevalent in many countries (e.g., Chen & Francis-Tan, 2021). The dearth of research in this area demonstrates a lack of attention to global contexts that may impact Black people in other countries, their trauma and subsequent coping strategies, and race-based experiences. Racialized individuals in American and Canadian society live in a unique context, with a violent and oppressive national history. Therefore, it is expected that different racial groups develop different coping responses in opposition to these maladaptive acculturation forces. Thus, it is reasonable to assume that there are social, cultural, and ethnic differences in regard to identifying the best strategies for coping with racism. For example, African Americans might not cope with racism in the same way as first-generation Black Caribbeans. However, these nuances are not taken into account in the current literature. For this reason, it is difficult to generalize these findings to persons racialized as Black globally because they do not include the majority of them. The topic of racially specific and effective coping strategies as an issue needs to be addressed in future research to provide a well-rounded and comprehensive view of how all people racialized as Black respond to racism.

Race and ethnicity were conflated in this study because most articles reviewed did not report these characteristics separately. Sex and gender were conflated for the same reasons. Relatedly, there is a lack of research that concentrates on the responses of Black LGBTQ+ people or other intersectionalities (i.e., class, disability, etc.) other than gender. Very few articles reviewed accounted for the challenge of living with intersectional identities. For example, the way queer Black Americans face racism might not be the same as cis-heterosexual African Americans people; therefore, this would be important to study and compare. Research demonstrates that the experienced reality for American Black women in regard to racism is different because it is also gendered. For this reason, it is very plausible that Black LGBTQ+ members’ responses to racist incidents have been influenced by their gender and/or sexuality (Spates et al., 2019). Without addressing these gaps in the literature, it will be impossible to quantify the magnitude, frequency, and range of responses of Black people to racism or the efficacy of their strategies or responses to racism. The global coping strategies of Black people are likely to be as varied as their experiences.

There were some limitations in distilling the data for the tables; specifically, in creating Table 3, the method used to sort the coping strategies into categories was based on the description provided in each article on that specific coping response. The articles were not always consistent in their use of terminology for various coping strategies, which made the process challenging, and there may certainly be room for refinement as more data are collected.

Although many studies drew connections between types of coping styles and well-being, most were descriptive, and even among those that were able to show statistical relationships between well-being and coping styles, almost none were designed such that directionality could be determined (for an exception, see D. M. Pittman & Kaur, 2018). This is clearly an area that would benefit from longitudinal and experimental research paradigms to quantify functionality.

Although seldom mentioned in the research literature, it is well-known that acts of artistic prowess, including but not limited to music, visual arts, and prose, can serve as positive, active problem-focused coping mechanisms (Stuckey & Nobel, 2010). This type of creative coping differs from the previously mentioned methods because it incorporates an act of creation. Here we refer to a self-affirmative, defiant act of beauty that serves to negate the message of worthlessness present in racist speech and thought patterns. Some of the major cultural treasures of history are creative acts that defy the message of racism with a message to the world reclaiming one’s own existence, proclaiming value, and ultimately making agency itself physical, with a tangible creation. In this way we have entire art forms collectively born out of anti-Black racism (e.g., jazz, blues, rap; Secundy, 1989; Stuckey & Nobel, 2010). Future studies should examine the efficacy of a broader repertoire of coping strategies.

Finally, this review excluded children. Although our literature search unearthed some research focused on responses of African American children and adolescents to racism that may be helpful in understanding the development of racism-related coping, this must be a topic for a separate review.

### Conclusion

A spectrum of coping strategies are being used by Black people to respond to their lived experiences of racism. These strategies encompass emotion-focused coping strategies such as religion and spirituality, as well as problem-focused coping strategies such as social support. Gender differences are evident in these coping responses, with Black women prioritizing spirituality and social support. There are commonalities between the coping strategies used by Black people for stressors that are emotional versus those that are physical, some of which may be race-specific, although self-care is clearly underutilized. Black individuals noticeably prefer to utilize active strategies when coping with racism, which helps to diminish the loss of agency that accompanies racism. Therapeutic methods should deemphasize coping strategies that reinforce notions of powerlessness in favor of more functional strategies that bring about growth and change. More research focused on outcomes for well-being is needed in this important area.

## Transparency

*Action Editor:* June Gruber

*Editor:* Laura A. King
